# A deep learning model using hyperspectral image for EUS‐FNA cytology diagnosis in pancreatic ductal adenocarcinoma

**DOI:** 10.1002/cam4.6335

**Published:** 2023-07-17

**Authors:** Xianzheng Qin, Minmin Zhang, Chunhua Zhou, Taojing Ran, Yundi Pan, Yingjiao Deng, Xingran Xie, Yao Zhang, Tingting Gong, Benyan Zhang, Ling Zhang, Yan Wang, Qingli Li, Dong Wang, Lili Gao, Duowu Zou

**Affiliations:** ^1^ Department of Gastroenterology Ruijin Hospital, School of Medicine, Shanghai Jiao Tong University Shanghai China; ^2^ Shanghai Key Laboratory of Multidimensional Information Processing East China Normal University Shanghai China; ^3^ Department of Pathology Ruijin Hospital, School of Medicine, Shanghai Jiao Tong University Shanghai China

**Keywords:** artificial intelligence, deep learning, endoscopic ultrasound‐guided fine‐needle aspiration, neural network models, pancreatic ductal carcinoma

## Abstract

**Background and Aims:**

Endoscopic ultrasonography‐guided fine‐needle aspiration/biopsy (EUS‐FNA/B) is considered to be a first‐line procedure for the pathological diagnosis of pancreatic cancer owing to its high accuracy and low complication rate. The number of new cases of pancreatic ductal adenocarcinoma (PDAC) is increasing, and its accurate pathological diagnosis poses a challenge for cytopathologists. Our aim was to develop a hyperspectral imaging (HSI)‐based convolution neural network (CNN) algorithm to aid in the diagnosis of pancreatic EUS‐FNA cytology specimens.

**Methods:**

HSI images were captured of pancreatic EUS‐FNA cytological specimens from benign pancreatic tissues (*n* = 33) and PDAC (*n* = 39) prepared using a liquid‐based cytology method. A CNN was established to test the diagnostic performance, and Attribution Guided Factorization Visualization (AGF‐Visualization) was used to visualize the regions of important classification features identified by the model.

**Results:**

A total of 1913 HSI images were obtained. Our ResNet18‐SimSiam model achieved an accuracy of 0.9204, sensitivity of 0.9310 and specificity of 0.9123 (area under the curve of 0.9625) when trained on HSI images for the differentiation of PDAC cytological specimens from benign pancreatic cells. AGF‐Visualization confirmed that the diagnoses were based on the features of tumor cell nuclei.

**Conclusions:**

An HSI‐based model was developed to diagnose cytological PDAC specimens obtained using EUS‐guided sampling. Under the supervision of experienced cytopathologists, we performed multi‐staged consecutive in‐depth learning of the model. Its superior diagnostic performance could be of value for cytologists when diagnosing PDAC.

## Graphical abstract

Accurate pathological diagnosis of pancreatic ductal adenocarcinoma (PDAC) poses a challenge for cytopathologists. Therefore, we aimed to develop a hyperspectral imaging (HSI)‐based convolution neural network (CNN) algorithm to aid in the diagnosis of pancreatic cytology specimens obtained using endoscopic ultrasonography‐guided fine‐needle aspiration/biopsy (EUS‐FNA/B). A total of 1913 HSI images were obtained from 33 benign pancreatic tissue samples and 39 PDAC specimens, and the model was trained on these images.

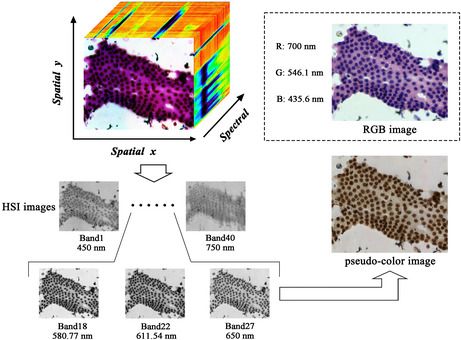

## 1 INTRODUCTION

Pancreatic ductal adenocarcinoma (PDAC) is a significant cause of cancer‐related mortality globally, with a poor prognosis and an increasing incidence such that it is likely to become the second leading cause of cancer death within 10 years.^1^, ^2^ Making an accurate pathological diagnosis of PDAC is essential for follow‐up management and treatment. Endoscopic ultrasonography‐guided fine‐needle aspiration/biopsy (EUS‐FNA/B) is considered to be a first‐line procedure for the pathological diagnosis of pancreatic cancer owing to its high accuracy. EUS‐FNA/B reportedly has a diagnostic sensitivity of 85%–92% and a specificity of 96%–98% for PDAC.^3^, ^4^ Moreover, it has been proven to be a feasible and safe technique with a complication rate of less than 1%.^5^, ^6^

The cytologic atypia and architectural distortion of PDAC are similar to that seen in chronic pancreatitis; specifically, the presence of atypical ductal epithelial cells might create confusion.^7^ The usual concern is that PDAC is clinically misinterpreted as reactive epithelial changes of pancreatitis. Crowded architecture, high nuclear to cytoplasmic ratio, irregular nuclear membranes, prominent nucleoli, and vacuolated cytoplasm are helpful in the specific diagnosis of pancreatic cancer.^8^ However, prominent nucleoli could be present in both reactive ductal epithelium of pancreatitis and PDAC, and this feature is not sufficient to distinguish between these two diseases.^9^ In addition, the presence of necrosis acts as a strong support for the diagnosis of PDAC. Whereas, the pathological smears of pancreatic pseudocyst with pancreatitis are often composed of necrosis and inflammatory debris, which breaks the above rule and makes it difficult for cytopathologists to differentiate from PDAC. Therefore, an accurate cytopathological diagnosis of PDAC is challenging for general or inexperienced pathologists.

Artificial intelligence (AI) based on deep learning models has been proved to assist in the diagnosis of cervical, thyroid, and pancreatic cancer, and has potential in for facilitating clinical diagnostic applications.^10^, ^11^, ^12^ Compared with pathologists, deep learning models have a shorter training period and a higher objectivity. The evaluation of pancreatic lesions using deep learning models was reported to result in highly accurate diagnoses.^12^, ^13^ However, all previous research was based on cytomorphological features under traditional optical microscopy followed by the identification of pancreatic diseases through deep learning. Momeni‐Boroujeni et al. reported the use of a multilayer perceptron neural network (MNN) to classify pancreatic specimens obtained from EUS‐FNA as benign or malignant in 2017, which is the first study that can be retrieved using FNA/FNB samples for cytological analysis.^12^ It finally achieved 77% accuracy for the atypical cases and more information might be required to reduce uncertainty regarding the likelihood of carcinoma. In 2022, Lin et al. reported an AI model which used to substitute manual rapid on site cytopathological evaluation during EUS‐FNA.^14^ It had good performance in detecting cancer cells, and presented an accuracy of 83.4% in the internal validation dataset and the similar result in the external validation dataset (88.7%). The implementation of above AI model could accelerate slide evaluation and reduce the waiting time for endoscopists. Hyperspectral imaging (HSI) is a new optical diagnostic technology that combines spectroscopy, it can measure the interaction of tissue and light through an HSI camera and further obtain spectral features that cannot be captured by conventional imaging modalities and provide more information for identification and differentiation.^15^ The specific spectral information reflects the chemical composition and content of different substances, which will change the optical and pathological properties of the tissue with the development of diseases and could be transformed into quantitative diagnostic information. However, the information produced by HSI is impossible to directly interpret by clinicians, and it often requires the help of computational algorithms, especially machine learning models. In recent years, HSI has been used in assisting diagnosis, micro‐environmental monitoring, and margin assessment of solid tumors through taking advantage of their composition‐specific spectral features.^16^, ^17^ HSI has been also proven to achieve promising results in the identification of diseases at the cytological level.^18^, ^19^ A study of the application of HSI model in distinguishing blood cells and it reached a great classification performance (Accuracy = 97.65%).^18^ However, there is not a published article to compare the classification accuracy of AI model to RGB images and HSI images at the same time, so it is difficult to determine whether the spectral information obtained by HSI will bring greater benefits to disease recognition.

Until now, the HSI features of PDAC cells obtained by EUS‐FNA/B and their application value in the diagnosis of PDAC have not been reported. In this article, we developed an HSI‐based convolution neural network (CNN) algorithm and compared the classification accuracy for RGB images and HSI images. The aim is to improve the diagnosis of pancreatic EUS‐FNA/B cytology specimens prepared using a liquid‐based cytology (LBC) method and to increase and advance the knowledge of pathologists.

## 2 MATERIALS AND METHODS

### 2.1 Case selection

In order to construct deep learning model and evaluate its diagnostic performance, the archives of the department of cytology were queried for pancreatic FNA specimens accessioned between January 2020 and January 2022 at a single medical center (Ruijin Hospital). Furthermore, the pancreatic FNA specimens accessioned between November 2022 and March 2023 were also collected as additional test cases to prove the generalization ability of the model. The complete demographic data of all cases are displayed in Appendix S1. According to the guidelines for pancreaticobiliary cytology from the Papanicolaou Society of Cytopathology, all cases of LBC‐prepared slides that were categorized as “negative for malignancy,” “atypical,” “suspicious for malignancy,” or “positive for malignancy” were included.^20^ For statistical purposes, suspicious FNA diagnoses were classified as positive for malignancy. All selected specimens had their respective LBC‐prepared slides reviewed by a second independent cytopathologist. For cancerous group, we included patients with histologically confirmed PDAC based on evaluation of resected specimens. For benign group, all cases were obtained from pseudotumoral chronic pancreatitis or autoimmune pancreatitis, which had histological diagnoses and underwent a follow‐up period of 6 months. The exclusion criteria were (1) EUS‐FNA/B specimens that were nondiagnostic, (2) all cases that have not been histologically confirmed, and (3) obtained regions of interest (ROI) were less than eight microscopic vision fields. Overall, a total of 72 patients were analyzed with histological diagnoses; 60 cases (32 cases were diagnosed as PDAC and 28 as benign cases) were selected for the training, validation and testing of the CNN algorithm, 12 cases (7 PDAC cases and 5 benign cases) were used to conduct additional test. This study protocol was approved by the ethics committee of Shanghai Jiao Tong University School of Medicine (No. (2022) Linlun‐213th) and was conducted in accordance with the World Medical Association (Declaration of Helsinki). Written consent was obtained from each participant before enrollment into this study.

### 2.2 Image acquisition and preprocessing

The hematoxylin–eosin‐stained LBC slides were scanned with an Olympus DP73 camera (Olympus Corporation) scanner, and the images were analyzed by two pathologists who have more than 10 years of professional experience. According to the Papanicolaou Society of Cytopathology guidelines proposed by Pitman et al., pathologists classified cases as PDAC or benign and manually delineated the ROIs on these images for further collection of hyperspectral images.^20^ Subsequently, the scanned slides were imported into QuPath (v.0.3.0, University of Edinburgh) for review^21^; the ROIs were then annotated by the pathologists (GLL and ZBY) and exported to the local drives.

**FIGURE 1:**
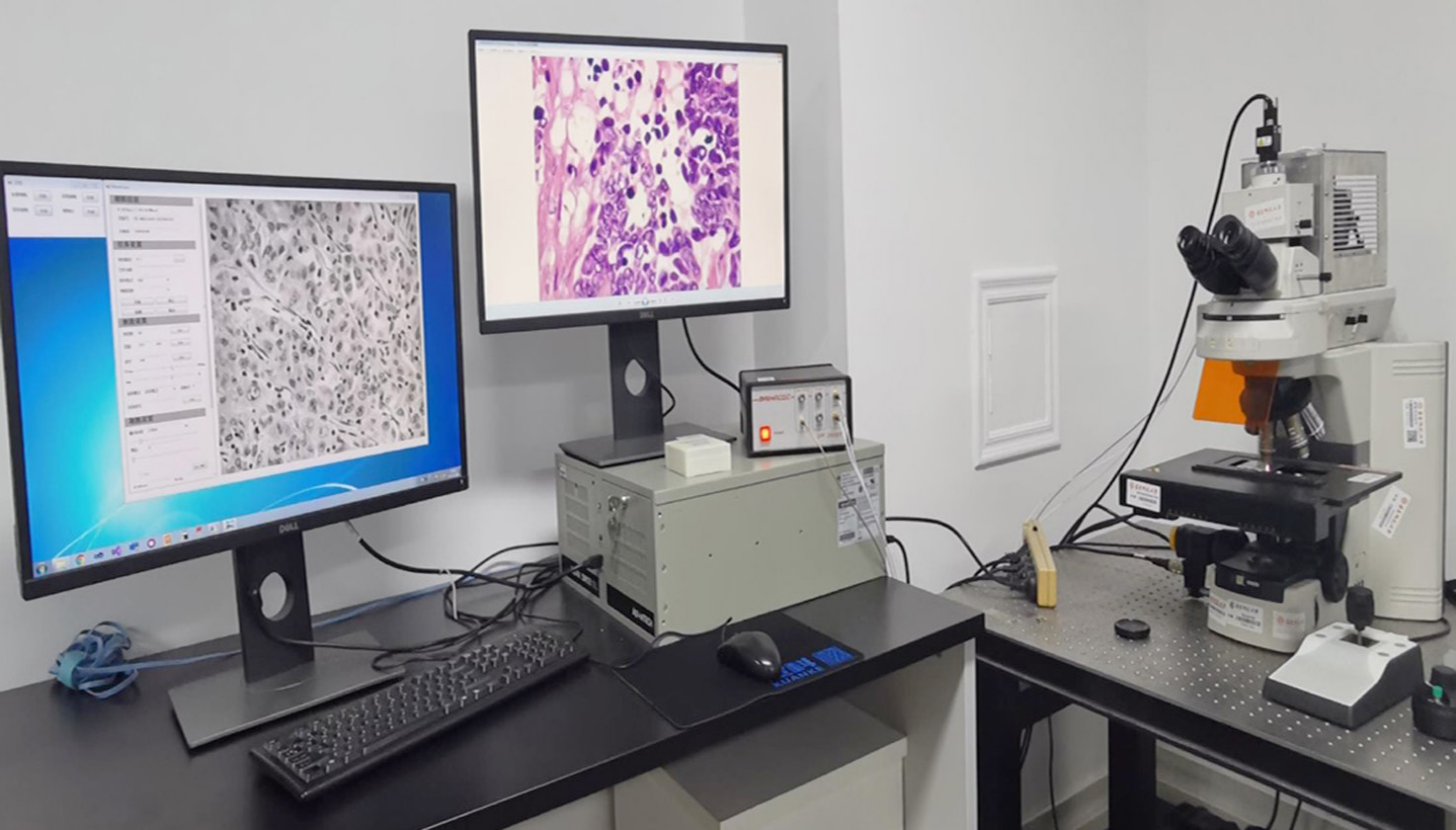
The hardware configuration of MHSI system.

**FIGURE 2:**
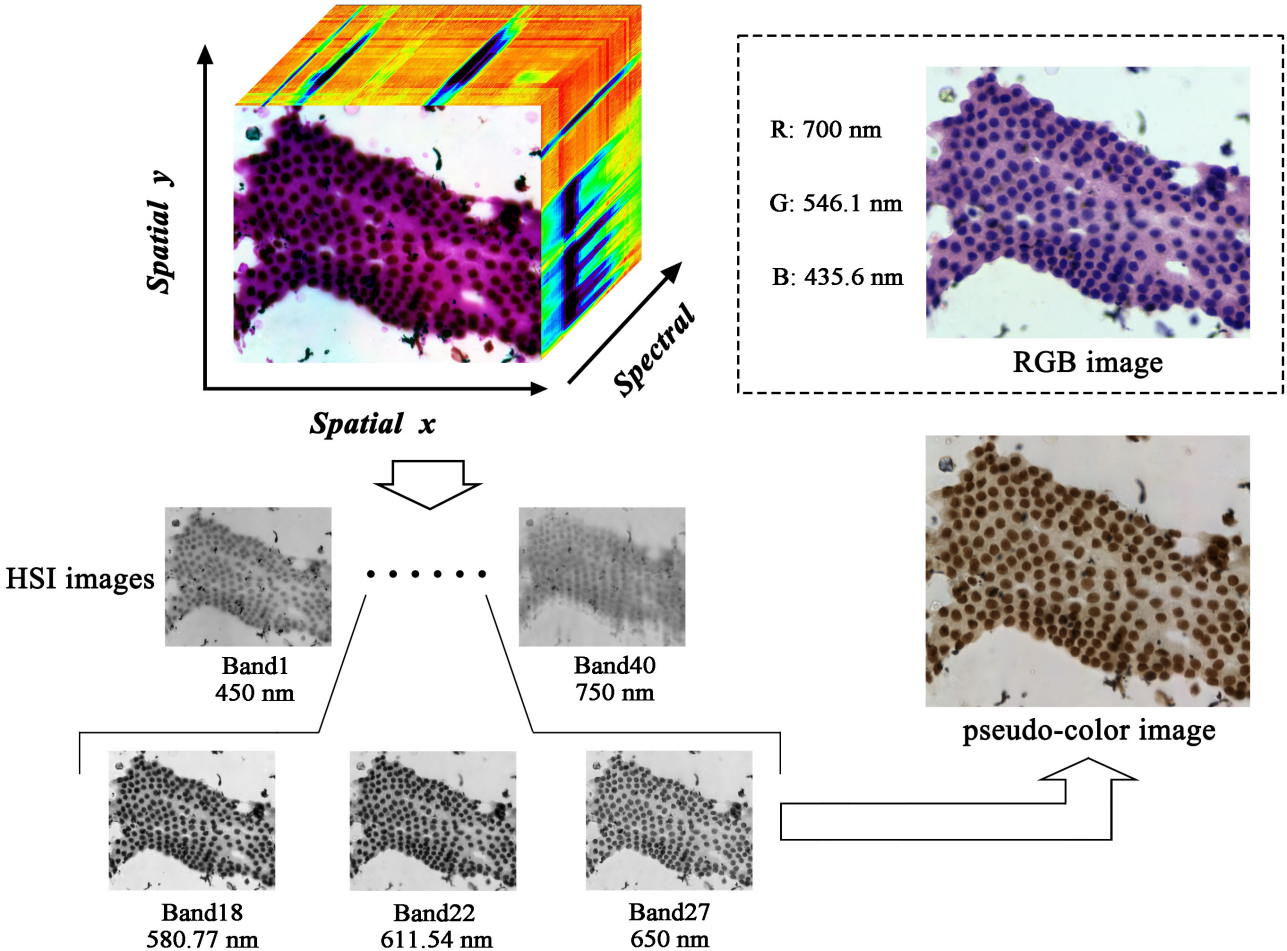
Microscopic hyperspectral image data cube and RGB image of the same field of view. The collected hyperspectral image data cube contained the images of 40 bands, three bands (Band18, Band22, and Band27) are selected to generate the pseudo‐color HSI image. Hematoxylin–eosin‐stained RGB image is also made up of the images of three bands (R: red band; G: green band; B: blue band).

The image information of the labeled slides was collected for hyperspectral pathology imaging using a homemade medical hyperspectral imaging (MHSI) system at the Shanghai Key Laboratory of Multidimensional Information Processing, MHSI system is shown in Figure 1.^22^ The system is based on an optical microscope with a color charge‐coupled device (CCD) camera from Omron, an acousto‐optic tunable filter (AOTF) from Brimrose, and a grayscale Complementary Metal‐Oxide‐Semiconductor (CMOS) camera from Omron installed in the upper part of the system. The hyperspectral data were in the spectral dimension of 450–750 nm with a band number of 40 bands (i.e., the intensity of light transmitted through the sample at 450, 457.5, 465 nm, etc.), and a spectral resolution of 7.5 nm. Before the image collection, MHSI system has been calibrated, including spatial and spectral calibration. In the process of image collection, the sample is placed on the carrier table and the halogen light source is mounted at the base. The whole system is a transmitted light imaging system. The acquisition of color and hyperspectral images is regulated by the reflector. When the reflector is pulled out of the optical path, the light propagates straight upward into the color CCD camera to obtain color images; when the reflector is inserted into the optical path, the light is reflected and passes through the AOTF into the grayscale CCD camera to obtain hyperspectral image data. Figure 2 displays microscopic HSI data cube, the RGB image and the pseudo‐color image with the same field of view. Compared with the RGB image, the microscopic HSI data cube contains more pathological information which is helpful for the diagnosis of PDAC.

The transmission of optics in the microscope and the detection sensitivity of the CCD camera might interfere with the hyperspectral images and cause data redundancy and noise. We used principal component analysis to select the components with the largest amount of information as the reference band. We first manually marked the whole cell of benign and PDAC in the HSI images, and a blank hyperspectral image was used as the reference image to eliminate any electronic instrument noise. Then, the nucleus of benign and PDAC were extracted and segmented by using a few‐shot GAN network in order to obtain the manual annotation masks and generate the characteristic spectra of benign and PDAC cells.^23^ There were great differences of spectral curves between benign and malignant cells in the spectral range from 530 to 620 nm, and a total of 20 consecutive bands were selected for the segmentation task; the remaining unselected bands were considered to be redundant and were excluded.

### 2.3 Development and generalization ability of the CNN algorithm

We adapted the ResNet architecture that can be directly applied to analyze HSI images and RGB images and has good generalization abilities for benign pancreatic tissues and PDAC.^24^ The collected pool of image dataset was used to develop the CNN algorithm, which obtained from 60 patients: 32 samples were categorized as PDAC, and 28 were categorized as benign cases. To ensure reliable results and fair comparisons, we used training set, validation set, and test set as the main method of the experiment and employed data augmentation consisting of variations in brightness, saturation, and rotation to boost the performance of the CNN. Specifically, we randomly separated the preprocessed hyperspectral data into three datasets according to the distribution of cell types: 60% for training, 20% for validation, and 20% for testing. Furthermore, 12 cases collected from November 2022 to March 2023 were defined as additional test set to prove the generalization ability of the model, including seven PDAC samples and five benign samples. Meanwhile, in order to realize the reproducibility of diagnostic performance, we fixed random seed before the model training. During the training stage, we expanded the training set, and the expanded dataset was used to construct the CNN model and minimize the loss function as much as possible. The validation set was used to evaluate the general error rate of the model, and to adjust the hyperparameter based on it. The classification accuracy of the test set was used as the performance of our classification algorithm. Finally, the accuracy of the CNN model for the additional test set and the test set was compared to evaluate its generalization. There was no overlap of patients between the different splits.

In general, a two‐phase deep learning algorithm was required to process the data. In phase one, we used the blank HSI image as the reference image to eliminate noisy data.^25^ The aim of this phase was to reduce the influence of electronic instrument noise so that we could obtain the significant characteristic spectra of the cytopathological specimens. The equation of image processing was as follows:

$$I_{\text{out}}=\frac{I_{\text{rawimage}}}{I_{\text{blank}}}$$

The second phase involved designing suitable architectures to handle natural RGB images and spatial‐spectral data cubes that are distinct from natural images. In the process, a vanilla CNN‐like model was applied as our backbone by adjusting the first convolutional layer. Then, a multilayer perceptron was set to map the features extracted from the CNN model to the final prediction. We set the initial learning rate to 1e^−05^. The maximum training epoch was 200 for each model and the training step stopped when training loss of test set no longer decreased.

On this basis, SimSiam algorithm was added into the HSI‐based CNN model to perform self‐supervised learning of image representations to further improve the model training efficiency and generalization ability. It achieved a higher accuracy without using negative sample pairs, when used in combination with ResNet architectures.^26^ Specifically, we adjust the first convolution layer of ResNet architecture to accommodate multi‐channel input images and pre‐train the whole architecture via SimSiam algorithm to improve generalization capabilities. Note, our proposed method could be adapted to almost all existing deep learning classification frameworks. All experiments were performed using Pytorch (Facebook's AI Research Lab [FIRE]), an open‐source machine learning library designed for deep learning and its relative applications, and conducted on a single NVIDIA GeForce RTX 3090 graphics processing unit (NVIDIA).

### 2.4 Explainability analysis

Attribution Guided Factorization Visualization (AGF‐Visualization) is a technique to visualize regions of important classification features that are identified by deep learning models.^27^ To investigate if the model was learning the correct features, we utilized the AGF‐Visualization technique to visualize potential pathological areas on the HSI images. AGF‐Visualization produced a class activation map for each HSI image through the final convolutional layer of ResNet architecture, it can highlight discriminative object regions for the class of interest and the part with the strongest activation was used for the model to make predictions. Specifically, we utilize the gradients to highlight the different importance of each location in the feature map for the class of interest. Through such operation, the fine‐grained details of the target cells can be effectively kept while the details in the background can be removed.

### 2.5 Statistical analysis

To evaluate the classification performance of the CNN model, the primary outcome measures included sensitivity, specificity, false‐negative rate (FNR), false‐positive rate (FPR), precision, recall, and the smoothness of receiver operating characteristic (ROC) curves, area under the ROC curves (AUC). The samples were divided reasonably by selecting the appropriate threshold and calculating the corresponding precision and recall to indicate how many model predictions are actually correct. AUC‐ROC curves are used as performance measurements for classification problems at various threshold settings. The ROC is a probability curve, and the circle on the ROC curve corresponds to a point with a threshold of 0.5; the area on the lower right of the ROC curve represents the AUC, which measures the separability, with a larger AUC indicating better performance. All statistical analyses were performed using Sklearn package in Python software (version 3.11.3, Python Software Foundation).

## 3 RESULTS

### 3.1 Study data

**FIGURE 3:**
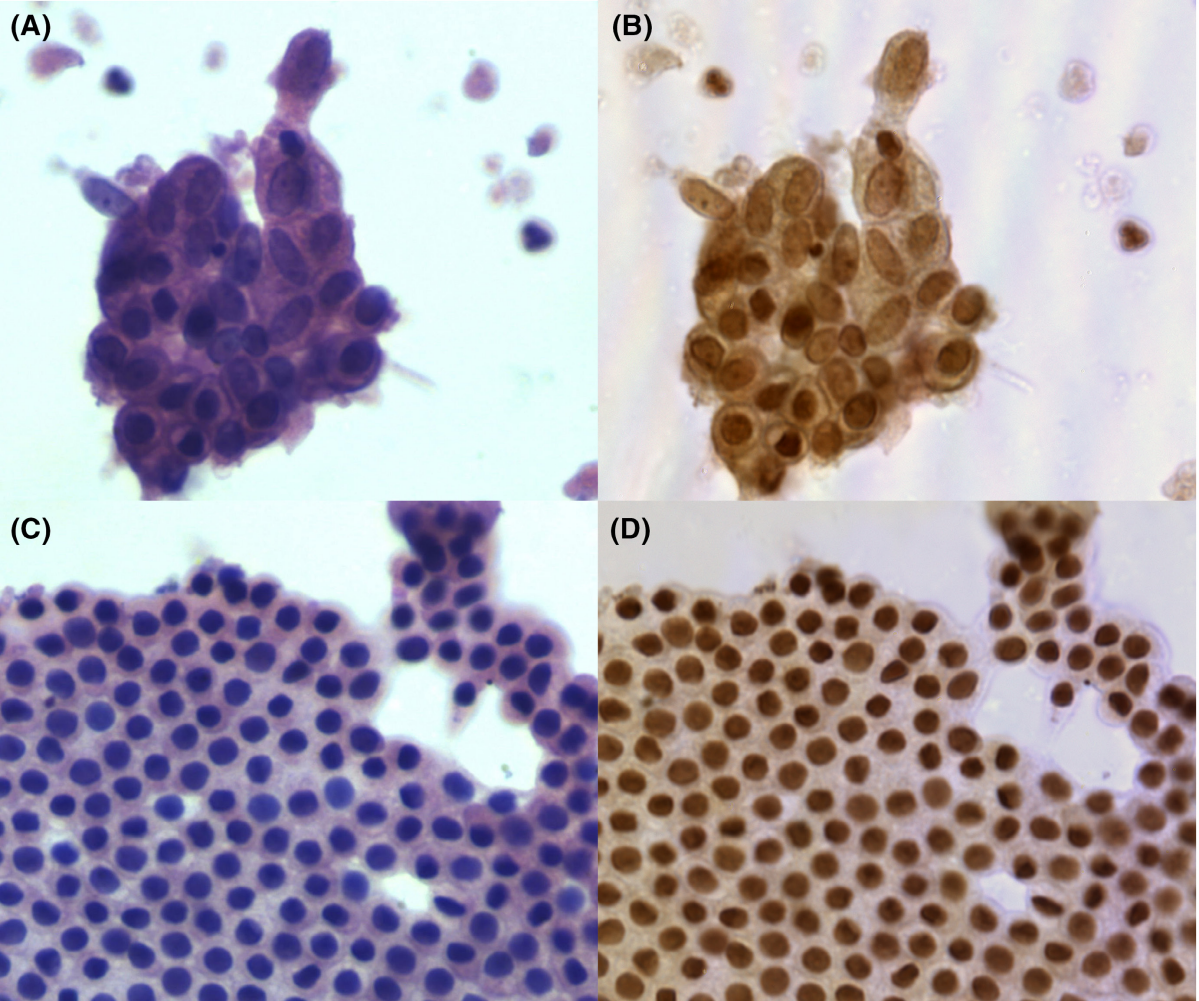
Comparison of hematoxylin–eosin‐stained RGB image and its corresponding HSI images of typical clusters of cells used for training. (A) Typical PDAC hematoxylin–eosin‐stained RGB image and (B) the corresponding HSI image of PDAC; (C) benign hematoxylin–eosin‐stained image and (D) its HSI image.

The aim of our study was to train a binary classification model to differentiate between benign pancreatic cells and PDAC cells. The study included 72 patients and the baseline characteristics are presented in Table 1. The mean age was 60.2 ± 13.2 years, and 47 (65.3%) patients were males. The mean size of lesions was 3.0 ± 1.1 cm and most of them were located in pancreatic head–neck (65.3%). Additionally, 40.3% of cytopathological diagnoses were positive for malignancy and 8.3% was suspicious for malignancy, 38.9% was negative and the rest were classified as atypical. A total of 1913 pairs of HSI and RGB images were obtained. The dataset used for developing the model consisted of 1025 effective scenes of images and they were exported from the MHSI system in two formats: HSI images, 512 × 612 pixels resolution, TIFF format, and RGB images, 2048 × 2448 pixels resolution, JPG format. RGB and HSI images were matched on the same field of view for qualitative comparison. Figure 3 displays an example of a hematoxylin–eosin‐stained RGB image and its corresponding HSI image of typical clusters of cells used for training. Due to the limited amounts of images, data augmentation (random cropping, flipping, rotation, scaling, and translation) was utilized to expand the dataset and a total of 1719 HSI images and corresponding RGB images were eventually collected. Among these scenes, 890 of them belonged to PDAC, and the rest ones were benign cases. Furthermore, 194 effective scenes of images were collected to prove the generalization ability of the model, including 101 PDAC images.

**TABLE 1:** Baseline characteristics of the training, validation, test, and additional test sets.

Baseline	Training set (*n* = 35)	Validation set (*n* = 13)	Test set (*n* = 12)	Additional test set (*n* = 12)	Total (*n* = 72)
Age (years), mean ± SD	60.6 ± 14.1	62.6 ± 15.7	56.6 ± 12.1	59.9 ± 8.3	60.2 ± 13.2
Sex, *n* (%)					
Male	23 (65.7%)	6 (46.2%)	10 (83.3%)	8 (66.7%)	47 (65.3%)
Female	12 (34.3%)	7 (53.8%)	2 (16.7%)	4 (33.3%)	25 (34.7%)
Size (cm), mean ± SD	3.1 ± 1.2	3.3 ± 0.9	2.8 ± 1.1	2.7 ± 1.0	3.0 ± 1.1
Location, *n* (%)					
Head/neck	25 (71.4%)	8 (61.5%)	6 (50.0%)	8 (66.7%)	47 (65.3%)
Body/tail	10 (28.6%)	5 (38.5%)	6 (50.0%)	4 (33.3%)	25 (34.7%)
Cytopathological diagnosis, *n* (%)					
Positive for malignancy	16 (45.7%)	5 (38.4%)	4 (33.4%)	4 (33.3%)	29 (40.3%)
Suspicious for malignancy	1 (2.9%)	2 (15.4%)	1 (8.3%)	2 (16.7%)	6 (8.3%)
Atypical	4 (11.4%)	2 (15.4%)	1 (8.3%)	2 (16.7%)	9 (12.5%)
Negative for malignancy	14 (40.0%)	4 (30.8%)	6 (50.0%)	4 (33.3%)	28 (38.9%)
Final diagnosis, *n* (%)					
PDAC	19 (54.3%)	8 (61.5%)	5 (41.7%)	7 (58.3%)	39 (54.2%)
Benign	16 (45.7%)	5 (38.5%)	7 (58.3%)	5 (41.7%)	33 (45.8%)

Abbreviation: PDAC, pancreatic ductal adenocarcinoma.

### 3.2 Spectrum features and differences between PDAC and benign cells

**FIGURE 4:**
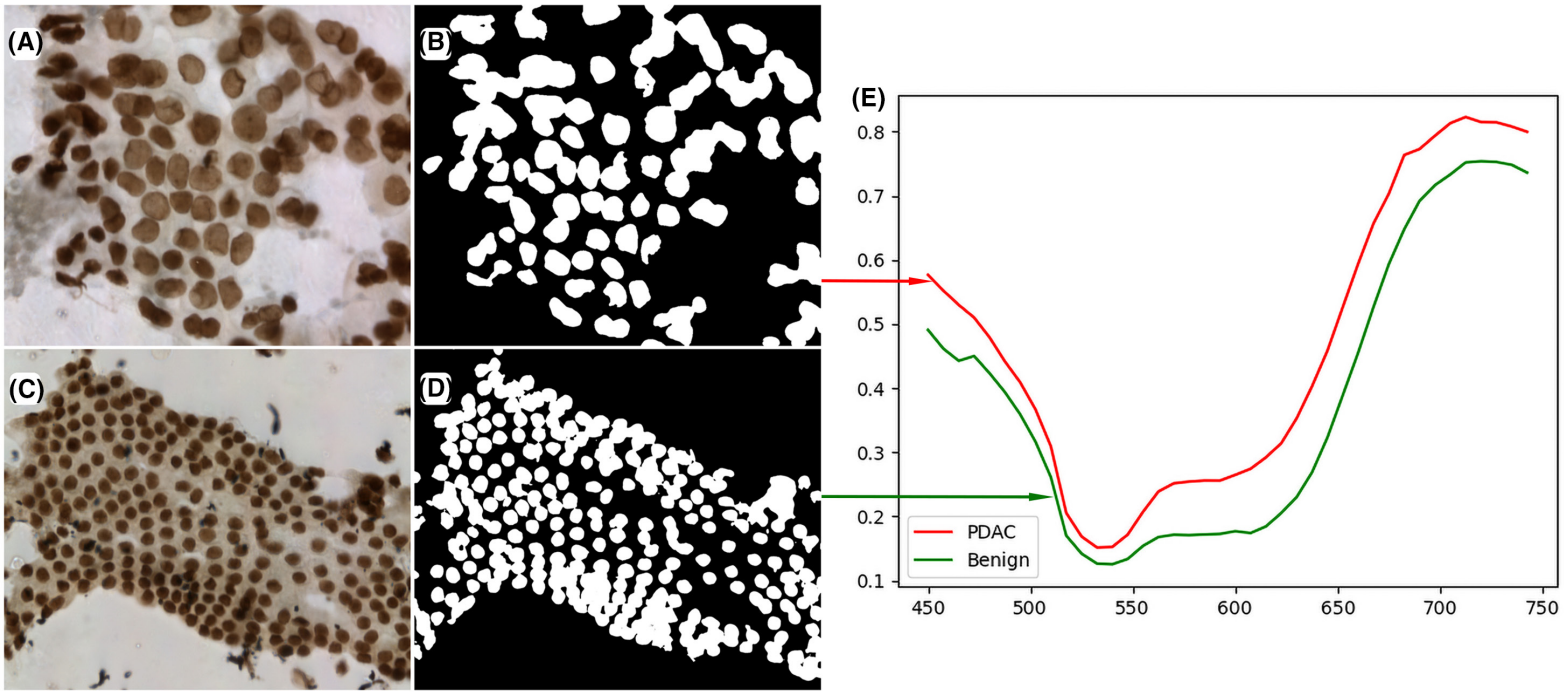
The representative spectrum features of benign pancreatic ductal/acinar cells and PDAC cells. (A) the pseudo‐color HSI images of PDAC cells; (B) the manual annotation masks of PDAC which are obtained by few‐shot GAN network; (C) the pseudo‐color HSI images of benign cells; (D) the manual annotation masks of benign; (E) The red curve represents the transmittance spectrum feature of PDAC in the hyperspectral images, and the spectrum feature of benign cells is the green curve.

The manual annotation masks of benign pancreatic ductal/acinar cells and PDAC cells were generated by using a few‐shot GAN network to process the pseudo‐color HSI images and the representative spectrum features of benign cells and PDAC cells are shown in Figure 4. The red curve represents the transmittance spectrum feature of PDAC in the hyperspectral images, and the spectrum feature of benign cells is displayed by the green curve. The typical spectral features of PDAC cells and benign pancreatic ductal/acinar cells are obviously different in the wavelength range of 530–620 nm. Therefore, different types of pancreatic cells could be classified by identifying their spectral and spatial features.

### 3.3 Comparison of the RGB‐based CNN and the HSI‐based CNN

By utilizing these image patches, we compared the performance between the RGB‐based CNN and the HSI‐based CNN model. Among the expanded datasets for the construction of the model, 890 images obtained from PDAC cases and 829 images from benign cases. The scenes of HSI images and their matching RGB images were first separated into three independent datasets, 1341 scenes for the training set, 177 for the validation set, and 201 for the test. With repeated input of HSI data and RGB data into the multilayer CNN, the training, and validation accuracies of CNN models were improved. For the validation set, the accuracy of ResNet18‐RGB was 91.72%, which was lower than ResNet18‐HSI with an accuracy of 93.22% (Table S1). For the test set, compared with ResNet18‐RGB that had an accuracy of 82.47%, ResNet18‐HSI achieved a higher accuracy of 88.05%. In addition, the sensitivity and specificity of ResNet18‐HSI were also higher than ResNet18‐RGB (Table 2). Therefore, one thing can be learned is the spectral information along with the spatial information make it easier for CNN models to distinguish PDAC and benign cells.

**TABLE 2:** Diagnostic performance of the CNN model.

Model	Accuracy (%)	Sensitivity (%)	Specificity (%)	Precision (%)	Recall (%)	FNR (%)	FPR (%)	AUC (%)
ResNet18	82.47	89.01	73.02	82.65	89.10	10.99	26.98	90.79
(RGB)								
ResNet18	88.05	94.25	83.33	81.18	94.25	5.75	16.67	95.91
(HSI)								
ResNet18	92.04	93.10	91.23	89.01	93.10	6.90	8.77	96.25
+SimSiam								
(HSI)								

Abbreviations: AUC, area under the ROC curves; FNR, false negative rate; FPR, false positive rate; ROC, receiver operating characteristic.

### 3.4 Diagnostic performance of the HSI‐based CNN model

**FIGURE 5:**
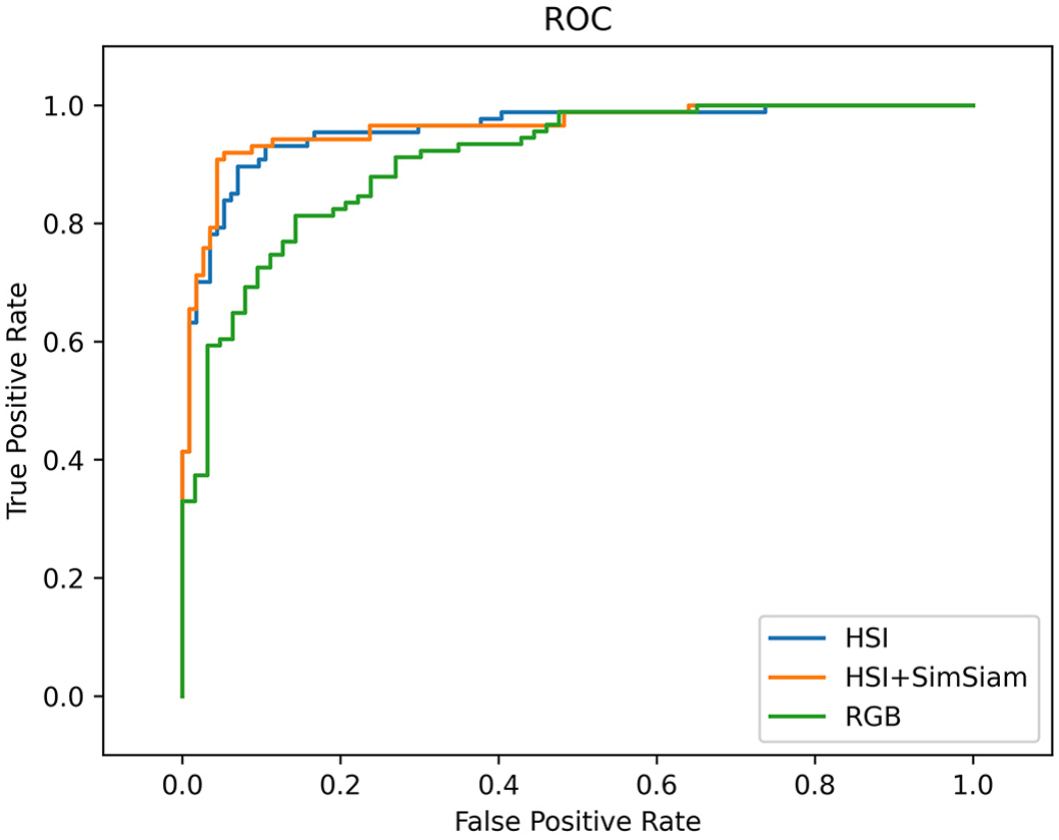
Comparison of the receiver operating characteristic (ROC) curve of CNN model at the image level. Green curve represents ResNet18‐RGB with an area under the curve [AUC] of 0.9079, blue curve indicates ResNet18‐HSI with AUC of 0.9591, and orange curve is ResNet18‐HSI‐SimSiam model with an AUC‐ROC performance of 0.9625.

We further employed SimSiam algorithm for ResNet18‐HSI model to analyzing existing sets of data augmentation, and evaluated the diagnostic performance of the HSI‐based models and calculated their relative metrics. Table 2 summarizes the metrics calculated for CNN models. Overall, the diagnostic performance of the ResNet18‐HSI‐SimSiam model was slightly higher than that of the purely ResNet18‐HSI model. The accuracy of the ResNet18‐HSI‐SimSiam model was 92.04%, and its sensitivity, specificity, FNR, and FPR for the diagnosis and differential diagnosis of benign cells and PDAC were 93.10%, 91.23%, 6.90%, and 8.77%, respectively. In contrast, the overall accuracy of the purely ResNet18‐HSI was 88.05%, its specificity was slightly lower and its sensitivity was similar to ResNet18‐HSI‐SimSiam. Furthermore, we used ROC curve to analyze the probability prediction of multi‐class classification. The ROC curve could balance the true positive rate and FPR in the prediction models with different probability thresholds, and the upper left corner of the plots was regarded as the true positive rate with a value of 1, whereas the FPR was 0. The value of the AUC of the ROC in our ResNet18‐HSI‐SimSiam model for the differential diagnosis of PDAC was 0.9625, whereas ResNet18‐HSI had an AUC‐ROC performance of 0.9591. Figure 5 demonstrates the ROC curves, and confusion matrices are shown in Tables 3 and 4.

**TABLE 3:** Confusion matrix for the true labels and predicted labels in ResNet18‐HSI.

Actual	Predict		
	PDAC	Benign	Total
PDAC	82	5	87
Benign	19	95	114
Total	101	100	201

Abbreviation: PDAC, pancreatic ductal adenocarcinoma.

**TABLE 4:** Confusion matrix for the true labels and predicted labels in ResNet18‐HSI‐SimSiam.

Actual	Predict		
	PDAC	Benign	Total
PDAC	81	6	87
Benign	10	104	114
Total	91	110	201

Abbreviation: PDAC, pancreatic ductal adenocarcinoma.

In addition, nine atypical cases were included in our study and five cases were eventually confirmed as PDAC. Our ResNet18‐HSI‐SimSiam model is also used to predict the benign or malignant outcomes of the atypical cases in all test sets. The accuracy of the ResNet18‐HSI‐SimSiam model for the images of the atypical cases was 83.02%, with its sensitivity and specificity were greater than 80% (Table S2 and S3).

### 3.5 Generalization ability of the CNN

In order to prove the generalization ability of our ResNet18‐HSI‐SimSiam model, we used ResNet18‐HSI‐SimSiam model to classify the additional test set. For the additional test set, a total of 194 images were collected. Overall, the diagnostic accuracy of the ResNet18‐HSI‐SimSiam model was 92.27%, which was similar to the 92.04% accuracy of the test set. Notably, the value of AUC of our model on the additional test was 0.9683, and it achieved a comparable result to the AUC of 0.9625 on the test set (Tables S2 and S4). Therefore, our ResNet18‐HSI‐SimSiam model showed generalizable and robust performance.

### 3.6 Computing performance of the CNN

Our ResNet18‐HSI‐SimSiam model could process the validation and test sets with an average speed of 0.144 s/frame. This translates to an approximate read rate of 6.94 frames per second.

### 3.7 Explainability of the model

**FIGURE 6:**
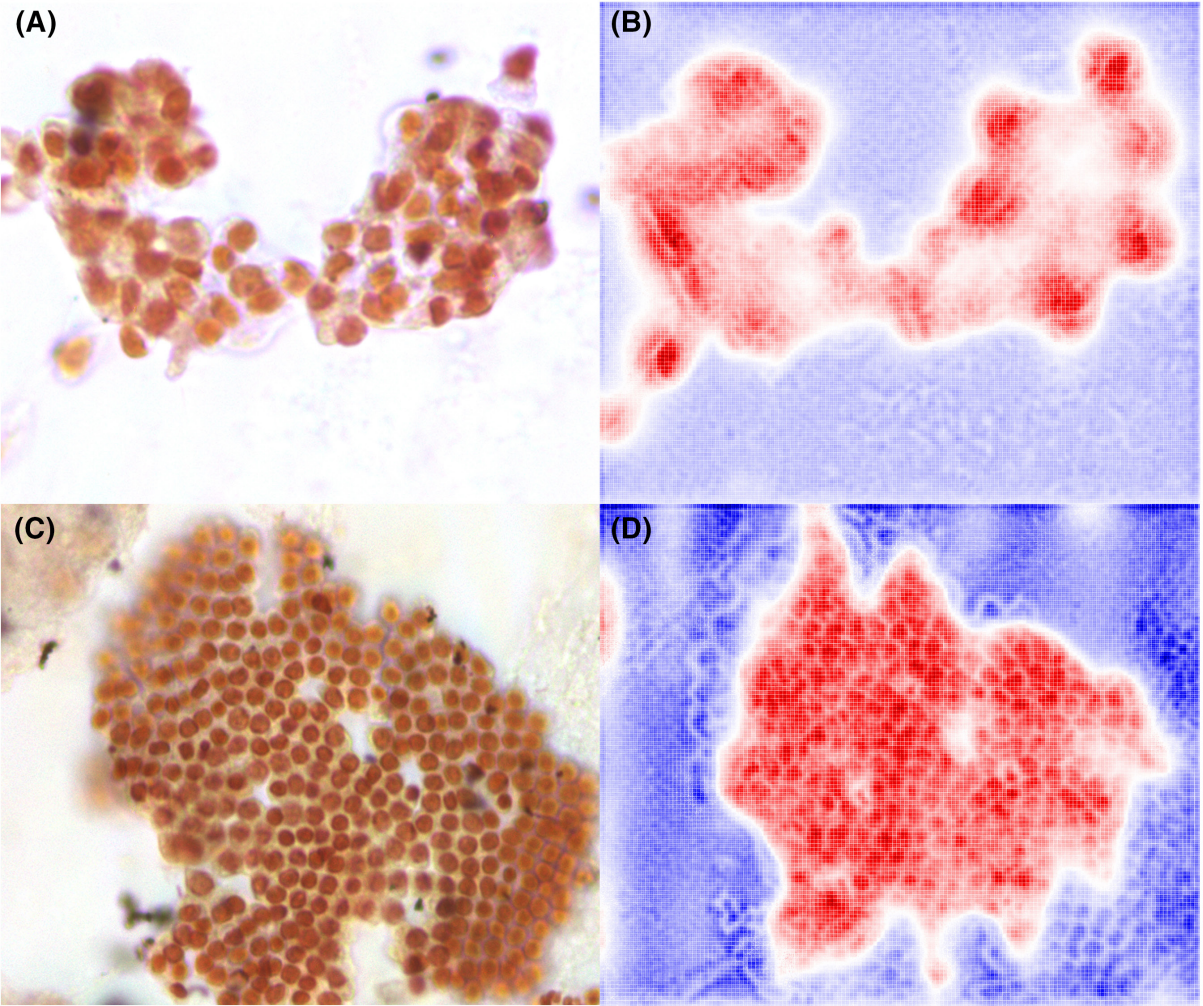
Visualization of two cases using AGF‐Visualization on HSI‐based model. (A) original HSI image of PDAC case; (B) class activation map using the AGF‐Visualization for PDAC case; (C) the HSI image of benign case and (D) class activation map of benign case. The high intensity area (red color) reflects the area of interest to our model.

The representative class activation maps generated by AGF‐Visualization are shown in Figure 6. Both the nuclei in benign cells and the nuclei in PDAC displayed the greatest level of activation, which is important for the model to make correct predictions. Therefore, we concluded that the classification of our model is based on morphological features rather than technical features.

## 4 DISCUSSION

Accurate pathological diagnosis of pancreatic specimens requires professional cytopathologists with sufficient experience; moreover, the rising incidence of pancreatic cancer is increasing the workload of pathologists.^28^ However, currently, cytopathologists are in short supply, and very few are proficient in pancreatic cytopathology, which poses a challenge for the cytological diagnosis of pancreatic cancer. Therefore, we developed a ResNet‐18 architecture with SimSiam using HSI images to diagnose pancreatic EUS‐FNA specimens prepared using an LBC method, and the model was validated to have good performance (AUC = 0.9625). AGF‐Visualization confirmed that the diagnoses were based on the features of tumor cell nuclei.

In recent years, LBC in cytological diagnosis has become increasingly popular as it reduces debris/lubricant contamination and the presence of blood compared with conventional smear cytology.^29^, ^30^, ^31^ LBC slides have a cleaner background and preserve the cytomorphological features of the cells from the specimen; thus, it could allow better discrimination of fine cellular details and achieve more efficient cytological evaluation and easier AI recognition.^32^ The cytology specimens of PDAC show an abnormal cell structure and arrangement under traditional optical microscopy. Abnormalities on the cellular level specifically include nuclear enlargement; the nuclei in ductal epithelial cell clusters vary in size and might show a three‐ to fourfold variability. In addition, the major criteria of PDAC include nuclear crowding or overlapping, irregular nuclear membranes, and irregular chromatin, and the minor criteria include nuclear enlargement, single epithelial cells, necrosis, and an increased number of cells in mitosis.^8^ According to the guidelines for pancreaticobiliary cytology from the Papanicolaou Society of Cytopathology, two or more of the major criteria or one major criterium with three minor criteria should be met to establish a diagnosis of PDAC, which poses a challenge to cytopathologists.^8^, ^20^

Owing to the improvement of deep‐learning technologies and computing power in recent years, AI has made remarkable progress in recognizing complex cytopathological images, and some technologies have matured into commercial products.^33^ A few studies have reported the application of AI in the cytopathological diagnosis of pancreatic cancer; however, the number of these investigations is limited, and most of them were based on traditional optical imaging technology that collects and processes information from spatial dimensions.^12^, ^13^ A retrospective study of 75 pancreatic FNA specimens evaluated the ability of a CNN based on traditional optical microscopy to distinguish benign cells from malignant pancreatic cytology specimens.^12^ It was demonstrated that there were significant differences in features such as contour, perimeter, and area between benign and malignant cells. With binary classification using these features, atypical cases could be categorized as benign or malignant with 77% accuracy.^12^ This result was not satisfactory for clinical application, and the diagnostic accuracy was suboptimal. HSI can obtain additional information about the spectral dimension of the images besides traditional optical imaging information and has been verified to be beneficial for cytopathological diagnosis.^18^ Our team previously designed an HSI‐based deep learning model for the identification of lymphoblasts in blood samples and demonstrated that the model assists with early acute lymphoblastic leukemia diagnosis.^19^ Compared with HSI, the information about spatial features provided by traditional optical images is insufficient to differentiate between lymphocytes and lymphoblasts. Therefore, the development of a novel algorithm is significant for maximizing the spatial‐spectral information of HSI data for cytopathological level diagnosis.

Based on the above limitations, the development of an ideal CNN model that can accurately distinguish PDAC and non‐cancerous cases at the cytological level might help to improve the ability of cytopathologists to diagnose atypical pancreatic cancer cells. Therefore, we designed a proof‐of‐concept study that aimed to explore the potential of an HSI‐based AI model for cytologically diagnosing PDAC. We first used HSI images to generate the transmittance curves of benign and PDAC cells. These two curves seem similar and the major difference seeking to be that the PDAC curve is shifted up (Figure 4E). We hypothesized that the origin of curve difference might be attributable to changes in the amount and arrangement of PDAC chromatin. PDAC is a genetic and epigenetic disease associated with rendered complex genome, and the most prevalent mechanism is polyploidy or whole‐genome doubling which involved the alteration in the DNA content.^34^ As previous literature reported, the change of DNA content affects the spectral shape, and it is sufficient to distinguish cancer cells with high DNA content from normal cells.^35^ In addition, the epigenetic changes in PDAC such as DNA methylation and histone post‐translational modification increase genomic instability.^36^ These changes could affect the absorbance of DNA or histone, which lead to differences in spectral curves.^36^, ^37^, ^38^ From the cytomorphology aspect, overlapping and crowding nucleus of PDAC cells and irregular chromatin distribution could interfere the reflected and transmitted light from tissue captured by HSI microscope, which might also affect the spectral transmittance curve of PDAC cells.^39^ Subsequently, our divided 1719 pairs of HSI and RGB images into training set, validation set, and test set for constructing CNN model, respectively and used the classification accuracy of test set to indicate diagnostic performance. We fixed random seed before the model training, which could realize the reproducibility of diagnostic performance. As shown in Table 2, ResNet18‐RGB with an accuracy of 82.47% was lower than our CNN model which were trained with HSI images. Consistent results could also be obtained from the validation set, the diagnostic accuracy was raised in ResNet18‐HSI (Table S1). Therefore, our HSI model is superior to a model trained on the exact same images captured using traditional optical scanning. In order to further improve the training efficiency of our HSI model, we employed SimSiam algorithm for ResNet18‐HSI model. Ultimately, it achieved an accuracy of 92.04%, sensitivity of 93.10%, specificity of 91.23%, and a high AUC ROC performance of 0.9625, which is comparable to previous models for predicting malignant tumors.^10^, ^40^ Furthermore, we used the additional test set to prove the generalization ability of our ResNet18‐HSI‐SimSiam model, the diagnostic accuracy and the value of AUC achieved comparable results to the test set (accuracy 92.27% vs. 92.04%; AUC 0.9625 vs. 0.9683).

Our study contains several highlights, and the primary one is that this is the first study on the application of an HSI‐based CNN model for image analysis of pancreatic diseases at the cellular level. We found that the typical spectral features of benign pancreatic ductal/acinar cells and PDAC cells are obviously different in the wavelength range of 530–620 nm. Second, we confirm the superiority of the HSI‐based CNN model by comparing HSI images with RGB images which captured by traditional optical scanning. Meanwhile, it also supports that the spectral features obtained by hyperspectral imaging techniques are helpful in pathological classification and could provide more information for identification and differentiation. Third, our model possesses the advantages of high accuracy, sensitivity, and specificity. We conducted a patient‐based analysis, adopted cross‐validation (training set, validation set, and test set), and achieved good results, which confirmed the effectiveness of the prediction model. Fourthly, our algorithm has excellent performance in image processing, with a reading rate of about 6.94 frames per second. This means that our model could be valuable in helping cytopathologists make more consistent diagnoses and reducing the workload. Overall, our model could support medical centers that lack experience in the diagnosis of cytological specimens of pancreatic lesions obtained using EUS‐FNA.

However, our work also has several limitations that should be considered. The first is that our model could only differentiate between benign pancreatic cells and PDAC cells. It cannot identify other pancreatic diseases such as pancreatic neuroendocrine tumor, pancreatoblastoma, solid pseudopapillary neoplasms, pancreatic acinar cell carcinoma, intrapancreatic metastases from other primary tumors, and other rare solid pancreatic lesions. In a follow‐up study, we aim to improve the model by increasing the types of diseases that can be identified. The second limitation is the small number of cases in our study. However, this limitation was partly offset because we were able to obtain 1913 HSI and RGB images through data augmentation. The third is that our study was a single‐center retrospective study, and a well‐designed multi‐center study is needed in the future to further evaluate the new technology to ensure that the collected data are representative and could enhance the credibility of the experimental evidence.

In conclusion, we developed and validated an HSI‐based model for diagnosing cytological pancreatic EUS‐FNA/B specimens. Under the supervision of experienced pathologists, we performed multi‐staged consecutive in‐depth learning of the model. AGF‐Visualization allow for human scrutiny to detect undesirable behavior in AI, and the increasing diagnostic performance of our model could help pathologists to identify PDAC and lay the foundation for further exploration of AI in this field in the future.

## AUTHOR CONTRIBUTIONS

**Xianzheng Qin:** Conceptualization (equal); data curation (equal); formal analysis (equal); investigation (equal); supervision (equal); validation (equal); writing – original draft (equal). **Minmin Zhang:** Conceptualization (equal); data curation (equal); formal analysis (equal); investigation (equal); supervision (equal); writing – original draft (equal). **Chunhua Zhou:** Conceptualization (equal); data curation (equal); formal analysis (equal); supervision (equal); writing – review and editing (equal). **Taojing Ran:** Data curation (equal); formal analysis (equal); supervision (equal); writing – original draft (equal). **Yundi Pan:** Data curation (equal); formal analysis (equal); supervision (equal); writing – original draft (equal). **Yingjiao Deng:** Data curation (equal); formal analysis (equal); methodology (supporting); software (equal); validation (equal); visualization (equal). **Xingran Xie:** Data curation (equal); formal analysis (equal); methodology (supporting); software (equal); validation (equal); visualization (equal). **Yao Zhang:** Data curation (equal); formal analysis (equal); supervision (equal). **Tingting Gong:** Formal analysis (supporting); supervision (equal); writing – review and editing (equal). **Benyan Zhang:** Formal analysis (equal); supervision (equal); validation (equal); writing – review and editing (equal). **Ling Zhang:** Formal analysis (equal); supervision (equal); writing – review and editing (equal); writing – review and editing (equal). **Yan Wang:** Formal analysis (equal); methodology (equal); writing – review and editing (equal). **Qingli Li:** Formal analysis (equal); methodology (equal); writing – review and editing (equal). **Dong Wang:** Formal analysis (equal); supervision (equal); writing – review and editing (equal). **Lili Gao:** Conceptualization (equal); data curation (equal); formal analysis (equal); supervision (equal); writing – review and editing (equal). **Duowu Zou:** Conceptualization (equal); data curation (equal); funding acquisition (equal); investigation (equal); project administration (lead); resources (equal); supervision (equal); writing – review and editing (equal).

## FUNDING INFORMATION

This work was supported by grants from the Science and Technology Commission of Shanghai Municipality (No. 21S31903500 and No. 21Y11908100).

## CONFLICT OF INTEREST STATEMENT

The authors made no disclosures.

## ETHICS STATEMENT

This study protocol was approved by the ethics committee of Shanghai Jiao Tong University School of Medicine (No. (2022) Linlun‐213th) and was conducted in accordance with the World Medical Association (Declaration of Helsinki). Written consent was obtained from each participant before enrollment into this study.

## Supporting information


Table S1.

Table S2.

Table S3.

Table S4.

Appendix S1.

## Data Availability

The datasets used and/or analyzed during the current study are available from the corresponding author on reasonable request.
